# Every message counts: scientific analysis of tuberculosis communication materials in Gujarat

**DOI:** 10.1186/s12889-026-27648-y

**Published:** 2026-05-11

**Authors:** Urvish Joshi, Zeel Sheth, Sharon Baisil, Bhavesh Modi, Sanjay Kini B., Sumit Unadkat, Sheetal Shrimali

**Affiliations:** 1 Department of Community Medicine, Narendra Modi Medical College, Ahmedabad, Gujarat India; 2 Department of Community Medicine, Swaminarayan Institute of Medical Sciences & Research, Kalol, Gujarat India; 3 Department of Community Medicine, Malankara Orthodox Syrian Church Medical College, Kolenchery, Kerala India; 4 Indian Council of Medical Research - National Institute of Occupational Health, Ahmedabad, Gujarat India; 5 Department of Community Medicine, Kasturba Medical College, Manipal Academy of Higher Education, Manipal, India; 6 Department of Community Medicine, Shri MP Shah Govt. Medical College, Jamnagar, India

**Keywords:** Tuberculosis (TB) communication, TB IEC materials, Content analysis, Behavior change frameworks, COM-B, Health communication evaluation, Cultural relevance, Message clarity, Gujarat, TB prevention and adherence

## Abstract

**Background:**

TB-IEC is central to India’s TB elimination efforts through print and digital outputs for care-seeking, adherence, prevention, and stigma reduction. However, IEC portfolios expand without checks on whether messages are behaviorally enabling, actionable, and culturally resonant. Gujarat has produced diverse TB-IEC through government channels, creating an opportunity to examine content alignment with behavior-change constructs. This study evaluates the content, behavioral fidelity, clarity, and cultural relevance of TB-IEC materials in Gujarat, and compares performance across issuer, language, and format.

**Methods:**

A cross-sectional content analysis was performed after IRB and State TB-Cell approvals on 375 TB-IEC items (2017–2025), compiled from state repositories, online media, and archives. Items were cataloged under National TB Elimination Programme (NTEP) thrust areas, coded using a 40-item checklist from seven behavioral and communication frameworks (COM-B, HBM, TPB, SCT, TTM, WHO-COMBI, and CDC-CCI; binary scoring), with two 5-point ratings for clarity and cultural adaptation. Descriptive and comparative analyses (chi-square tests, t-tests, one-way ANOVA, correlations) were performed in MS Excel and SPSS version 25 (*p* < 0.05).

**Results:**

Materials were primarily print-based (84.5%), followed by video (14.4%) and audio (1.1%), with posters comprising 68.0%. Items were issued by state and central agencies in Gujarati (52.3%) and Hindi (39.5%), targeting the general public (91.2%). Mean Total Behavioral Breadth was 11.41/40. Topics covered symptoms (44.0%) and diagnosis (53.6%), while prevention topics were less common (preventive therapy 4.3%; vaccination 0.3%); stigma reduction appeared in 13.6%. Items were assessed for calls to action, credibility cues, audience testing signals, skill demonstrations, and barrier-addressing content. Format analysis showed significant differences in clarity and action-cue ratings between video and print, with format differences in adherence and social acceptance themes. Cultural adaptation and clarity ratings varied by issuer and language, while Total Behavioral Breadth remained consistent across issuers.

**Conclusions:**

Gujarat’s TB IEC output during 2017–2025 was mainly print-based, with limited prevention and stigma content across formats, languages, and issuers. These findings support framework-guided IEC audits, rebalancing toward prevention and stigma reduction, and audience-tailored messaging. Expanding effective formats with clear action cues, alongside pre-testing and monitoring, may strengthen TB communication products.

**Supplementary Information:**

The online version contains supplementary material available at 10.1186/s12889-026-27648-y.

## Introduction

Tuberculosis (TB) remains one of the most pressing public health challenges in India, accounting for over a quarter of the global burden [1]. Despite significant biomedical advancements in diagnostics and treatment, the epidemic is sustained by complex social determinants, including stigma, delayed health-seeking behavior, and suboptimal treatment adherence [2]. Recognizing that biomedical interventions alone are insufficient to achieve the ambitious goal of TB elimination by 2025, the National Strategic Plan (NSP) 2017–2025 has explicitly positioned Information, Education, and Communication (IEC) and Social and Behavior Change Communication (SBCC) as central pillars of the elimination strategy (Central TB Division, 2017 [3]. Effective communication is not merely supportive but essential for generating demand for services, ensuring adherence to long-term treatment regimens, and fostering the social norm change required to dismantle stigma.

In response to this mandate, government health agencies and development partners have produced a vast array of IEC materials-ranging from traditional print posters to digital video campaigns-aimed at diverse populations. However, the sheer volume of output does not guarantee effectiveness. A persistent challenge in public health communication is the ‘science-practice gap’, where materials are often developed based on intuition or administrative checklists rather than evidence-based behavioral science [4]. While materials may be technically accurate, they often fail to address the underlying psychological and structural barriers that prevent individuals from acting on that information. The prevailing communication landscape in many high-burden settings is characterized by directive, biomedical messaging that prioritizes ‘telling’ over ‘enabling’, often neglecting the nuances of health literacy, cultural relevance, and behavioral capability [5].

Globally, the efficacy of health communication is increasingly evaluated through rigorous behavioral frameworks. Models such as the Capability-Opportunity-Motivation-Behavior (COM-B) system, the Health Belief Model (HBM), and the Theory of Planned Behavior (TPB) provide structured lenses to assess whether a communication product successfully addresses the antecedents of behavior change [6–8]. Similarly, operational frameworks like the WHO Communication for Behavioral Impact (COMBI) and the CDC Clear Communication Index offer standardized metrics for strategic planning [9, 10]. Despite their validation in other domains, these frameworks have rarely been applied to the systematic evaluation of TB communication in India. This study addresses this gap by presenting the first large-scale, multi-format scientific assessment of government-issued TB IEC materials in Gujarat. By examining a corpus of 375 items against a composite 40-point codebook, this research moves beyond a simple inventory to a diagnostic evaluation of the state’s communication strategy within the context of the National Strategic Plan [3].

This study aimed to systematically evaluate the scientific quality, behavioral fidelity, and cultural adaptability of government-issued TB IEC materials in Gujarat, India. Specifically, it sought to quantify the presence of theory-based behavioral constructs, assess the clarity and contextual relevance of communication outputs, and compare effectiveness across media formats, issuing authorities, and thematic focus areas to inform future evidence-based communication policies.

## Methods

### Study design and setting

A descriptive cross-sectional content analysis was conducted to evaluate Tuberculosis (TB) Information, Education, and Communication (IEC) materials developed and disseminated in the state of Gujarat, India. Gujarat, a high-burden TB state in western India with a robust public health infrastructure, serves as a critical setting for assessing the quality of health communication strategies. The study period covered materials produced and circulated between January 2017 and July 2025, aligning with the implementation phase of the National Strategic Plan for Tuberculosis Elimination (2017–2025) [3].

The study is reported in accordance with the STROBE (Strengthening the Reporting of Observational Studies in Epidemiology) guidelines for cross-sectional studies (see Additional_File _1).

## Materials

### Sampling and selection protocol

A systematic search was conducted for materials published between January 2017 and August 2025 across official government portals (NTEP, MoHFW, Gujarat Health Department), social media repositories, and physical archives at the State TB Cell. Retrieved items (*N* = 412) underwent digital de-duplication and quality screening. We excluded technical guidelines for providers, administrative circulars, and low-resolution drafts. The final eligible corpus (*n* = 375) was cataloged in a Master Log. Detailed harvesting protocols, digital verification steps, and exclusion criteria are provided in Additional File 2.

### Data collection and coding procedures

#### Development of the codebook

To ensure a rigorous assessment, we developed a 40-point coding tool synthesizing constructs from seven established frameworks: the COM-B model [6], Health Belief Model (HBM) [7], Theory of Planned Behavior (TPB) [8], WHO COMBI [9], CDC Clear Communication Index [10], Social Cognitive Theory (SCT) [11], and Transtheoretical Model (TTM) [12]. The codebook assessed both theoretical alignment (e.g., presence of ‘susceptibility’ or ‘norms’) and execution quality. The complete codebook, including variable definitions and scoring protocols, is provided in Additional File 3.

For instance, the COM-B domain contributed six binary-coded items, such as the presence of instructions that build physical capability (e.g., demonstrating how to wear a mask) or messaging that fosters reflective motivation (e.g., appeals to family protection) [6]. In contrast, the WHO COMBI framework contributed eight items aligned with strategic planning processes, including evidence of message pre-testing or monitoring metrics [9].

Each item was scored as 0 (absent) or 1 (present). No differential weightage was applied to individual items; every construct was treated as equally significant for the behavioral impact of the material. Consequently, the total possible score for behavioral breadth was 40. The variation in the number of items per framework (e.g., 10 for CDC Index vs. 3 for TPB) reflects the granularity required to assess those specific domains rather than a weighting of importance. Procedural frameworks like the CDC Index and WHO COMBI require a more detailed checklist to evaluate execution quality (e.g., checking for font size, bullet points, and logos separately), whereas theoretical models like TPB are captured by broader psychological constructs [8–10]. This scoring logic ensures a balanced assessment of both the content (theoretical alignment) and the form (communication quality) of the materials.

#### Cataloguing and inventory management

A centralized ‘Master Log’ was created to inventory every procured item. Each file was assigned a unique alphanumeric identifier (e.g., TB_P_001) to ensure traceability. To ensure a distinct dataset, duplicate files were identified using digital fingerprinting and visual verification, retaining only the highest-resolution versions. This systematic cataloguing allowed for the precise exclusion of ineligible items and provided a stable denominator for analysis.

#### Addition of quality assessment scales

In addition to the 40-point checklist which measured the presence of content, two supplementary 5-point Likert scales were integrated to assess the quality of execution [13]. These were distinct from the binary codebook and were added to capture nuances that a simple checklist might miss:

1. Global Clarity Score (1–5):

Rated the overall visual accessibility and legibility of the material (1 = Cluttered/Unreadable; 5 = High contrast, ‘Picture Superiority’). This was included to differentiate between a poster that technically contains a message versus one that communicates it effectively to a lay audience.

2. Global Cultural Adaptation Score (1–5):

Rated the degree of contextual relevance to the Gujarat setting (1 = Generic/Foreign context; 5 = Highly localized with regional dialects, attire, and setting). This was added to measure the depth of ‘localization’ beyond simple translation, aligning with the principle that culturally resonant messages are more persuasive.

#### Coding process and reliability

The coding process was conducted in three phases to ensure rigor and minimize subjective bias.

##### Phase 1: pilot testing and calibration

The initial codebook was pre-tested on a randomly selected pilot subsample of 30 items (10% of the initial corpus). This phase aimed to identify ambiguities in item phrasing and ensure a shared understanding of theoretical constructs among the research team. Discrepancies in scoring during the pilot were discussed, leading to refinements in the codebook definitions (e.g., clarifying the distinction between ‘susceptibility’ and ‘severity’ in visual depictions).

##### Phase 2: independent coding

Following calibration, the full corpus of 375 materials was coded. To manage the volume while maintaining accuracy, the coding was primarily conducted by the principal investigator, with a trained co-investigator independently coding a random 10% subset of materials from each format category (print, video, audio). For video and audio materials, coders were instructed to review the content multiple times-first for overall messaging and tone, and subsequently for specific behavioral constructs-pausing and replaying as necessary to ensure no subtle cues were missed. Print materials, which constituted the largest segment of the corpus, underwent a meticulous, multi-pass review process. Each item was first assessed for its primary visual impact and layout hierarchy, determining the dominant message conveyed by headlines and imagery. A second, granular review scrutinized fine print, logos, and footer text to identify subtle credibility markers, helpline numbers, and institutional attributions that might otherwise be overlooked. This rigorous examination ensured that even minimalist formats like stickers or gadget branding were accurately scored for essential constructs such as ‘Cues to Action’ and ‘Evidence Credibility’.

##### Phase 3: reliability assessment and consensus

Inter-rater reliability was assessed on a stratified random sample of 38 items (10.1% of the corpus), comprising 32 print, 5 video, and 1 audio material. Reliability for the 40-item binary behavioral checklist was evaluated using Cohen’s Kappa (𝛋), while the ordinal 5-point scales (Clarity and Cultural Adaptation) were assessed using Intraclass Correlation Coefficients (ICC; two-way mixed effects, absolute agreement, single measures). A pre-determined acceptance threshold of 𝛋 > 0.75 and ICC > 0.75 was established. Discrepancies were resolved through a consensus meeting between the two coders to generate the final dataset. The average inter-rater reliability was found to be strong (Mean kappa = 0.88; Mean ICC = 0.91), with detailed domain-wise metrics provided in Additional_File_4 as Table S2. Inter-Rater Reliability Audit.

### Data analysis

Data were entered into MS Excel and analyzed using SPSS version 25 (IBM Corp, Armonk, NY) [14]. Descriptive statistics (frequencies, percentages) were used to characterize the corpus by format, issuer, and language. Thematic coverage was mapped against the 14 thrust areas of the National Strategic Plan: Symptoms, Diagnosis, Treatment Adherence, Infection Control, TPT (Preventive Therapy), Benefits (DBT), Ni-kshay Mitra (Community Support), Stigma Reduction, Comorbidities, General Awareness, Vaccination, TB-COVID Bidirectional Screening, Branding, and Drug-Resistant TB (DR-TB) [3]. CDC Clear Communication Index (referred to as ‘Clarity Index’ for brevity).

To assess scientific quality, mean scores were calculated for Total Behavioral Breadth (range 0-40) and for each theoretical sub-index. Comparative analyses were performed to test study hypotheses. Due to the limited sample size of audio materials (*n* = 4), this subgroup was excluded from inferential statistical comparisons to satisfy normality assumptions, though retained for descriptive reporting. To interpret the scientific quality scores, ‘Efficacy’ was operationalized as the theoretical potential to influence behavior based on framework fidelity, rather than clinical impact. To interpret the central tendency of the scores, Mean Total Behavioral Breadth was contextualized using tertiles: Low (< 13 points), Moderate (13–26 points), and High (> 26 points). Consequently, Independent Samples t-tests were used to compare mean clarity scores between Print and Video formats, and between Central and State issuers. Effect sizes (Cohen’s d) were calculated to assess the magnitude of observed differences, interpreted as small (0.2), medium (0.5), and large (0.8). Pearson’s correlation coefficient (r) was calculated to examine the relationship between visual clarity scores and scientific breadth. To facilitate visual comparison across heterogeneous scales in the radar chart (Fig. 2), scores were normalized to a percentage scale (0–100%) by dividing the mean group score by the maximum possible score for that index (e.g., Mean HBM Score / 6 × 100). Quantitative results are reported as Mean (Standard Deviation [SD]). To estimate the magnitude of observed differences, 95% Confidence Intervals (CI) for mean differences and effect sizes (Cohen’s d) were calculated. P-values are reported exactly, with *p* < 0.05 considered statistically significant.

To enhance transparency and reproducibility, the complete item-level dataset has been deposited in a public repository (Figshare), while the full codebook, STROBE checklist, and reliability statistics are provided as Supplementary/additional Files.

### Ethical considerations

Institutional Review Board (IRB) approval was obtained. (Ref: NaMoMC/IRB/2025/242). Administrative permission to access and analyze the IEC materials was granted by the State Tuberculosis Officer, Government of Gujarat. As the study involved the analysis of public domain materials with no human participants, informed consent was not applicable.

## Results

### Corpus characteristics

The study analyzed 375 IEC materials, predominantly comprising print formats (84.5%), with video (14.4%) and audio (1.1%) constituting the remainder (Table 1). Production sources were balanced between State (49.3%) and Central (43.2%) government agencies. Vernacular content predominated, with Gujarati appearing in 52.3% of materials, followed by Hindi (39.5%). Audience segmentation was minimal; 91.2% of materials targeted the general public rather than specific subgroups (Table 1).

**Table 1:** Descriptive profile of the analyzed IEC corpus (*N* = 375)

Variable	Category	Frequency (*n*)	Percentage (%)
Format	Print	317	84.50%
	Video	54	14.40%
	Audio	4	1.10%
Issuer Category	Central Govt (GoI / MoHFW / CTD)	162	43.20%
	State Govt (GoG / Health Dept)	185	49.30%
	Partner / NGO (WHO, Union, etc.)	28	7.50%
	Media / Corporate / Other	0	0.00%
Language	Gujarati	196	52.30%
	Hindi	148	39.50%
	English	16	4.30%
	Bilingual / Multilingual	15	4.00%
Target Audience	General Public	342	91.20%
	Patients / Caregivers	25	6.70%
	Providers / Other	8	2.10%

Data derived from master inventory log after de-duplication. Categories are mutually exclusive. Issuer Category definitions: Central Govt (MoHFW, CTD); State Govt (Gujarat Health Dept, State TB Cell); Partner/NGO (WHO, The Union, private partners). Language refers to the primary text/audio language; Bilingual indicates equal use of two languages

Among print materials, posters were the dominant sub-format (68.0%), while video content was distributed between short-form (< 1 min; 42.6%) and long-form productions (57.4%) (Table 2).

**Table 2:** Frequency distribution of sub-formats within print and video categories

Main Format	Sub-Format	Frequency (*n*)	Percentage (of Total *N*)	Percentage (within Format)
Print (*n* = 317)	Poster	255	68.00%	80.40%
	Leaflet / Brochure / Booklet	32	8.50%	10.10%
	Hoarding / Banner	15	4.00%	4.70%
	Sticker / Gadget Branding	10	2.70%	3.20%
	Wall Painting	5	1.30%	1.60%
Video (*n* = 54)	Short Video (< 1 min)	23	6.10%	42.60%
	Long Video (> 1 min)	31	8.30%	57.40%
Audio (*n* = 4)	Radio Spot / Jingle	4	1.10%	100.00%

Percentages within format calculated based on n_{print}=317 and n_{video}=54. Short videos include Reels, YouTube Shorts, and TVCs < 60 s

Within video formats, duration did not significantly impact efficacy; short videos (< 1 min) performed comparably to longer videos on clarity (*p* = 0.482) and actionability (*p* = 0.803) (Table 3).

**Table 3:** Comparative efficacy of video materials by duration (*n* = 54)

Metric	Short Videos (< 1 min) (*n* = 23) Mean(SD)	Long Videos (> 1 min) (*n* = 31) Mean(SD)	t-value	*p*-value
Global Clarity Score (1–5)	4.17 (0.8)	3.97 (1.0)	0.71	0.482
Action Index (/2)	1.65 (0.5)	1.61 (0.6)	0.25	0.803
HBM Index (/6)	2.43 (1.1)	2.74 (1.3)	-0.91	0.367
Total Breadth Score (/40)	11.83 (4.5)	14.19 (5.8)	-1.58	0.12

Analysis via Independent Samples t-test. Values are Mean ± SD. Differences in clarity and actionability between short (*n* = 23) and long (*n* = 31) formats were not statistically significant (*p* > 0.05)

### Thematic landscape and content focus

**Fig. 1:**
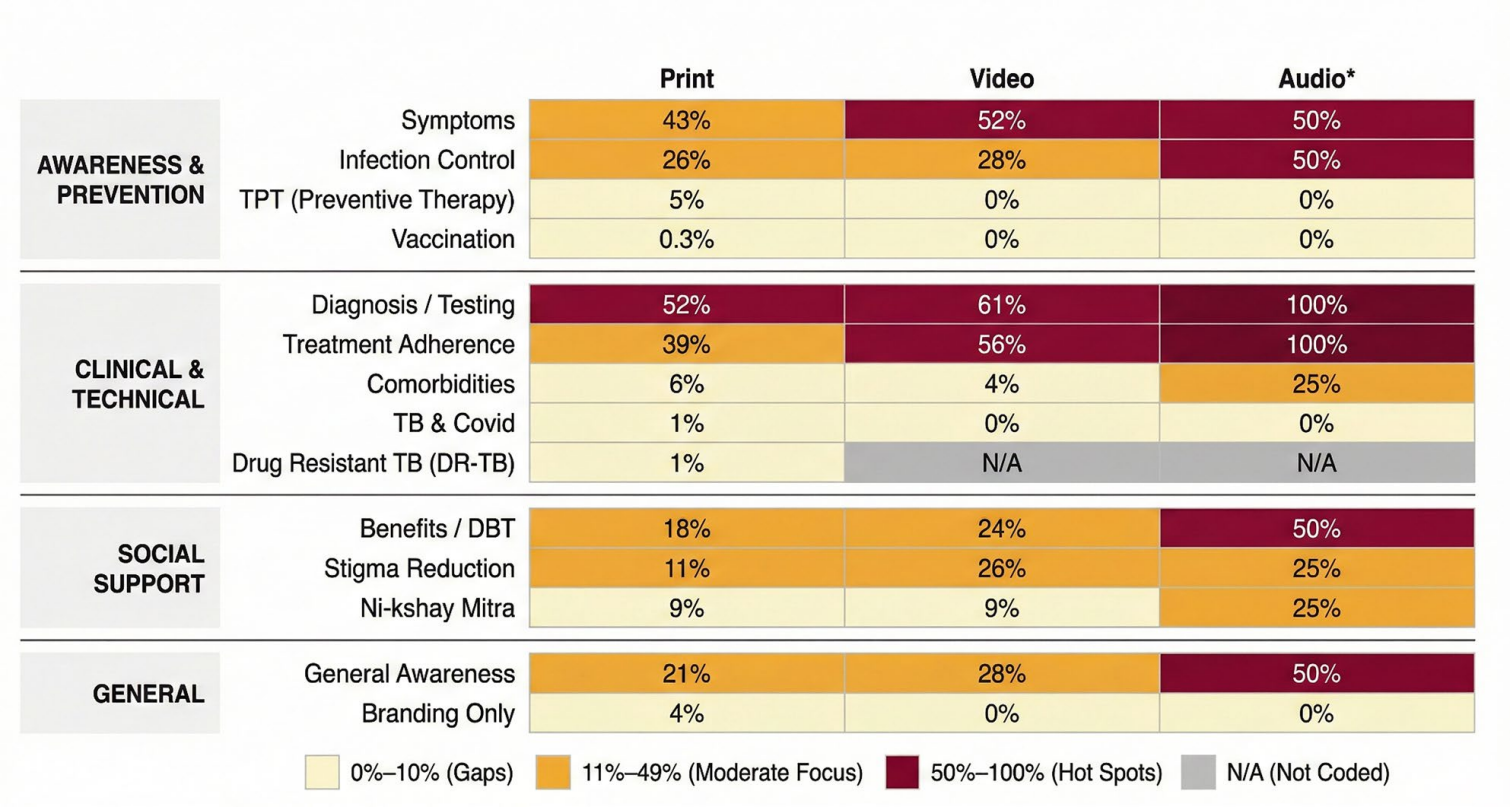
Thematic coverage of National Strategic Plan thrust areas across IEC formats. Note: Data represent the percentage of materials within each format (N_print = 317, N_video = 54, N_audio = 4) containing specific themes. p-values are from Chi-square test of independence, indicating significant associations between format and theme presence (Treatment adherence, *p* = 0.009; Social acceptance, *p* = 0.008). Shading intensity corresponds to prevalence frequency

Diagnosis (53.6%) and symptoms (44.0%) were the most saturated themes, whereas preventive strategies such as TPT (4.3%) and vaccination (0.3%) were negligible (Fig. 1). Social drivers of the disease were less visible, with stigma reduction appearing in only 13.6% of the corpus. However, format-specific analysis indicated that dynamic media were significantly more likely to address complex behavioral themes: video/audio materials covered treatment adherence (*p* = 0.009) and social acceptance (*p* = 0.008) more frequently than static print materials (Fig. 1).

### Scientific alignment and behavioral gaps

Overall adherence to behavioral frameworks was low-to-moderate, with a mean Total Behavioral Breadth score of 11.41 out of 40 (Table 4).

**Table 4:** Scientific alignment with behavioral frameworks and quality dimensions

Theoretical Domain (Index)	Max Possible Score	Mean Score (SD)	Median (IQR)	Interpretation
Total Behavioral Breadth	**40**	**11.41 (5.1)**	**11.0 (8.0–14.0)**	Low-Moderate Depth
Quality Dimensions				
Global Clarity Score	5	3.65 (1.1)	4.0 (3.0–4.0)	Good Visual Accessibility
Global Cultural Score	5	3.55 (1.3)	3.0 (3.0–5.0)	Generic to Adapted

Values represent central tendency (*N* = 375). Total Behavioral Breadth is the sum of 40 binary items (Present = 1, Absent = 0). Quality Dimensions (Clarity/Cultural) are scored on independent 5-point Likert scales and are not included in the Total Breadth sum. *SD* Standard Deviation, *IQR* Interquartile Range

Materials aligned strongest with the CDC Clear Communication Index (48.8% utilization) and Health Belief Model (35.8%), but showed minimal application of systemic frameworks like WHO COMBI (4.9%) (Table 5).

**Table 5:** Utilization rates of specific behavioral theories and frameworks

Behavioral Framework	Mean Score (SD)	Max Score	% Utilization	Key Constructs Assessed
1. CDC Clear Communication Index [10]	4.88 (1.9)	10	48.80%	Message Placement, Language, Visuals
2. Health Belief Model (HBM) [7]	2.15 (1.3)	6	35.80%	Susceptibility, Severity, Barriers, Cues
3. COM-B Model [6]	2.03 (0.9)	6	33.80%	Capability, Opportunity, Motivation
4. Transtheoretical Model (TTM) [12]	0.88 (0.5)	3	29.30%	Pre-contemplation, Action, Maintenance
5. Social Cognitive Theory (SCT) [11]	0.62 (0.7)	4	15.50%	Modeling, Reinforcement, Reciprocal Det.
6. Theory of Planned Behavior (TPB) [8]	0.45 (0.6)	3	15.00%	Attitudes, Norms, Control
7. WHO COMBI [9]	0.39 (0.6)	8	4.90%	Strategy, Pre-testing, Monitoring

% Utilization calculated as (Mean Score / Max Possible Score) × 100. Higher percentages indicate greater reliance on that specific theoretical framework in the design of the materials

Construct-level gap analysis revealed a directive, authority-based communication style; while 90.4% of items included active calls to action and 88.5% carried credibility markers, only 9.3% demonstrated physical skills (e.g., mask usage) and 14.1% addressed barriers such as side effects. No materials contained explicit evidence of pre-testing with the target audience (Table 6).

**Table 6:** Construct-level gap analysis: Strengths and weaknesses

Critical Behavioral Construct	Frequency (%)	BCC Implication
Strengths		
1. Q32 Call to Action (Active Verb)	90.40%	Directive Clarity: Explicit instruction.
2. Q40 Credibility (Logos)	88.50%	Institutional Trust: Visual authority via Govt emblems.
3. Q02 Psychological Capability (Facts)	85.30%	Knowledge Building: Focus on TB facts/symptoms.
Weaknesses		
1. Q28 Pre-Testing Evidence	0.00%	User Validation: No evidence of field-testing.
2. Q30 Monitoring Indicators	0.30%	Accountability: Lack of evaluation metrics.
3. Q01 Physical Capability (Skills)	9.30%	Skill Deficit: Low demonstration of ‘how-to’.
4. Q10 Perceived Barriers	14.10%	Empathy Gap: Fears rarely addressed.

Descriptive frequencies (*N* = 375). Selected items represent the constructs with the highest and lowest prevalence rates across the corpus

### Comparative assessment of quality

#### Format

**Fig. 2:**
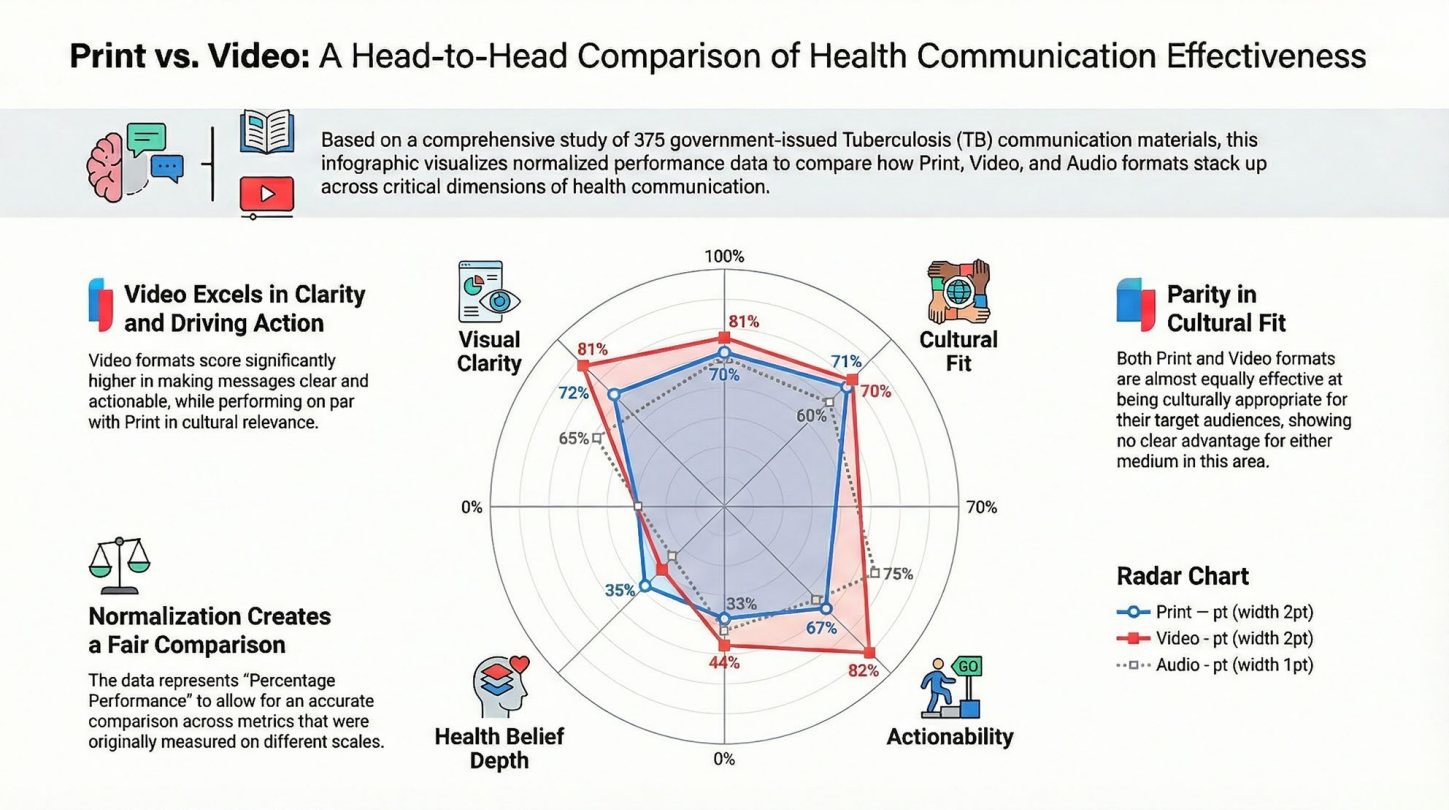
Comparative behavioral and quality profile of IEC materials by media format. Note: Radar axes represent normalized performance percentages (calculated as Mean Score ÷ Maximum Possible Score × 100) for Global Clarity, Cultural Tailoring, Action Index, and HBM Index. Video materials (*n* = 54) demonstrated significantly higher scores in Clarity (*p* = 0.004) and Action cues (*p* = 0.001) compared with Print (*n* = 317), determined via one-way ANOVA

Video materials scored significantly higher than print in Global Clarity (4.06 vs. 3.58; t = 3.12, *p* = 0.004), demonstrating a medium effect size (Cohen’s d = 0.46). Similarly, video formats provided significantly more actionable cues than print (*p* = 0.001, d = 0.52). While audio materials were analyzed descriptively (*n* = 4), they were excluded from statistical significance testing due to insufficient sample size (Table 7; Fig. 2).

**Table 7:** Quality and Actionability by Medium (Format Bias Check)

Metric	Print (*n* = 317)Mean (SD)	Video (*n* = 54)Mean (SD)	Mean Difference (95% CI) (Video - Print)	*P*-value	Effect Size (Cohen’s d)
Global Clarity Score (1–5)	3.58 (1.1)	4.06 (0.9)	0.48 (0.18 to 0.77)	0.004	0.46 (Medium)
Action Index (0–3)	1.34 (0.6)	1.63 (0.5)	0.29 (0.12 to 0.46)	0.001	0.52 (Medium)
HBM Index (0–6)	2.08 (1.3)	2.61 (1.2)	0.53 (0.16 to 0.90)	0.006	0.42 (Small-Med)
Cultural Tailoring (1–5)	3.56 (1.4)	3.52 (0.9)	-0.04 (-0.35 to 0.27)	0.575	0.03 (Negligible)

Comparisons represent Independent Samples t-tests between Print and Video formats. Audio materials (*n* = 4) were excluded from inferential testing due to insufficient sample size. SD: Standard Deviation; CI: Confidence Interval; HBM: Health Belief Model. Comparisons exclude Audio materials due to sample size limitations (*n* = 4). All scores normalized to a 0–100% scale or reported on their original Likert metrics as specified

#### Issuer and language

A strong localization effect was observed.

State Government materials (Mean = 4.29, SD = 1.0) achieved significantly higher Cultural Tailoring scores compared to Central Government materials (Mean = 2.83, SD = 1.0), with a mean difference of 1.46 (95% CI: 1.25–1.67). This difference represented a large effect size (d = 1.46), confirming stronger contextual relevance in state-produced content (t=-13.62, *p* < 0.001) (Table 8).

**Table 8:** Comparison of quality and content metrics by issuing authority

Metric	Central Govt (MoHFW/CTD) (*n* = 162) Mean (SD)	State Govt (Gujarat) (*n* = 185) Mean (SD)	Mean Difference (95% CI) (State - Central)	*P*-value	Effect Size(Cohen’s d)
Global Cultural Score (1–5)	2.83 (1.0)	4.29 (1.0)	1.46 (1.25 to 1.67)	< 0.001	1.46 (Large)
Global Clarity Score (1–5)	3.39 (1.1)	3.84 (1.1)	0.45 (0.22 to 0.68)	< 0.001	0.41 (Small-Med)
Total Breadth Score (0–40)	10.99 (4.9)	11.69 (5.3)	0.70 (-0.38 to 1.78)	0.203	0.13 (Negligible)

Statistical significance determined via Independent Samples t-test. 95% CI does not cross zero for significant variables. Comparisons exclude Audio materials due to sample size limitations (*n* = 4). All scores normalized to a 0–100% scale or reported on their original Likert metrics as specified

This advantage extended to linguistic clarity, where Gujarati materials (M = 3.91, SD = 1.1) scored significantly higher than Hindi counterparts (M = 3.34, SD = 1.1), with a mean difference of 0.57 (95% CI: 0.32–0.82; *p* < 0.001) (Table 9).

**Table 9:** Impact of language on visual clarity and cultural relevance

Language	Mean Clarity Score (1–5)	Mean Cultural Score (1–5)
Gujarati	3.91	4.35
Hindi	3.34	2.65
English	3.69	2.63
Bilingual	3.53	3.2
*p*-value (ANOVA)	< 0.001*	< 0.001*

Analysis via One-way ANOVA. Significant differences (*p* < 0.001) indicate that language choice influences perceived clarity, with vernacular materials scoring highest

#### Target audience

Segmentation significantly influenced scientific depth. Materials designed for Patients/Caregivers exhibited higher Total Behavioral Breadth (13.56 ± 4.5) compared to those for the General Public (11.22 ± 5.1; *p* = 0.041). However, visual clarity and cultural tailoring scores did not differ significantly by target audience, indicating that while patient-focused materials were more informative, they were not necessarily better designed or more culturally adapted (Table 10).

**Table 10:** Impact of target audience on quality and content depth

Metric	General Public (*n* = 342) Mean (SD)	Patients/Caregivers (*n* = 25) Mean (SD)	Providers/Other (*n* = 8)	F-value	*p*-value
Global Clarity Score (1–5)	3.64 (1.1)	3.88 (0.9)	3.50 (1.4)	0.61	0.543
Global Cultural Score (1–5)	3.54 (1.3)	3.64 (1.1)	3.63 (1.5)	0.14	0.867
Total Behavioral Breadth (0–40)	11.22 (5.1)	13.56 (4.5)	12.88 (6.2)	3.22	0.041*

Analysis via One-way ANOVA comparing means across audience groups (General Public, Patients/Caregivers, Providers). Bold p-values* indicate statistical significance (*p* < 0.05). Patient materials showed significantly higher scientific breadth

### Interaction of design quality and content depth

Analysis of content specificity revealed a functional trade-off. While multi-thematic materials naturally yielded higher total breadth scores due to their wider scope (*p* < 0.001), focused mono-thematic items maintained a robust Action Index (1.18/2.0). This indicates that while mono-thematic materials demonstrated functional efficacy through a robust Action Index (Table 11), their overall performance on comprehensive theoretical depth remained lower than that of multi-thematic materials.

**Table 11:** Thematic depth and efficacy by content focus

Content Type	*n*	Mean Total Score (/40)	Mean HBM Score (/6)	Mean Action Index (/2)
Mono-thematic	68	9.07	1.54	1.18
Multi-thematic	307	11.93	2.29	1.43
*p*-value		< 0.001*	< 0.001*	0.002*

Analysis via Independent t-test. ‘Mono-thematic’ items (n = 68) address a single thrust area; ‘Multi-thematic’ (*n* = 307) cover ≥ 2 areas. Action Index serves as a control for functional efficacy despite total score disparity

Correlation analysis revealed a moderate positive relationship between Global Clarity and Total Behavioral Breadth (*r* = 0.44). Notably, no correlation was observed between Cultural Tailoring and Behavioral Breadth (*r* = 0.03), suggesting that high cultural adaptation does not inherently guarantee comprehensive scientific content (Table 12).

**Table 12:** Pearson correlation matrix of quality and content variables

Variables	Global Clarity Score	Global Cultural Score	Total Behavioral Breadth
1. Global Clarity Score	1		
2. Global Cultural Score	0.37*	1	
3. Total Behavioral Breadth	0.44*	0.03	1

*Significant at *p* < 0.01. Findings indicate a moderate positive correlation between design clarity and scientific breadth (*r* = 0.44)

## Discussion

While India’s National Tuberculosis Elimination Programme (NTEP) has extensively documented the reach of its communication campaigns, the scientific quality and behavioral fidelity of these materials remain largely unexamined. Previous audits in low- and middle-income countries (LMICs) have typically been limited to single-format evaluations (e.g., newspapers or posters only) or qualitative reviews of small samples [15, 16]. Rarely has a study integrated multiple behavioral frameworks to assess the full spectrum of IEC outputs across print, video, and audio platforms. By bridging a critical evidence gap, this study establishes a novel baseline for understanding how well state-level communication aligns with elimination goals. The findings reveal a communication landscape that has successfully established institutional authority and biomedical literacy but remains constrained by a ‘curative’ bias, a directive rather than enabling tone, and a significant lag in adopting digital-first strategies [17]. While the corpus demonstrates strong adherence to basic clarity principles—as evidenced by high scores on the CDC Clear Communication Index—it exhibits critical gaps in content designed to address the social determinants and messaging intended to facilitate the ‘physical capability’ required for prevention.

### Aligning communication with evolving policy priorities

A defining characteristic of the analyzed corpus is its strong focus on biomedical themes, specifically symptoms (44.0%) and diagnosis (53.6%). This reflects the program’s historical success in raising awareness about active case finding. As the National Strategic Plan (2017–2025) shifts focus toward TB elimination, there is a timely opportunity to expand the communication portfolio to include preventive strategies like Tuberculosis Preventive Therapy (TPT), which was present in only 4.3% of the current materials (Fig. 1) [3]. Expanding the narrative from ‘cure’ to ‘prevention’ would align communication outputs with global best practices observed in other high-burden settings, where IEC strategies are evolving to support latent TB management [15].

From a theoretical perspective, the heavy reliance on the CDC Index and HBM (Table 5) suggests a strategy that prioritizes information delivery (susceptibility) over social modeling or norm-shaping. While current materials effectively elevate ‘Perceived Susceptibility’ by listing symptoms, the limited content on stigma reduction (13.6%) and social support neglects the ‘Perceived Barriers’ construct. Consequently, the communication style remains directive rather than enabling; while 90.4% of items include calls to action, only 9.3% demonstrate the ‘Physical Capability’ (e.g., skills) required for prevention (Table 6) [6]. This reflects a ‘science-practice gap’ where materials establish authority but do not adequately scaffold the complex behavioral changes required for elimination [18, 19].

To build on the existing foundation of authority, future materials could incorporate more empathetic elements. Currently, only 14.1% of materials address perceived barriers such as side effects or logistical challenges (Table 6). Furthermore, a significant opportunity exists to enhance ‘Physical Capability’ (COM-B). While materials often instruct users to ‘wear a mask’, only 9.3% included visual demonstrations of the correct technique. Moving from telling to teaching is a proven strategy to bridge the intention-behavior gap [18]. By increasing the frequency of skill-building content and role-modeling (SCT), the program can further empower the community to take effective preventive action. The overall low scientific breadth score of 11.41/40 (Table 4) underscores the need for deeper, multi-dimensional content that goes beyond basic awareness [19].

### The utility of focused messaging (Nudges)

The study assessed the trade-off between comprehensive education and focused ‘nudges’. Analysis of content specificity (Table 10) revealed that while mono-thematic materials (e.g., a sticker focused solely on stigma) naturally scored lower on total scientific breadth (*p* < 0.001), they maintained a robust Action Index (1.18/2.0). This validates the functional utility of simple, focused creatives. Not every IEC material needs to be a comprehensive encyclopedia; simple prompts that trigger a specific action (e.g., “Call 1800.“) play a vital role in the Action stage of the Transtheoretical Model [12]. A balanced IEC portfolio should arguably retain these high-frequency ‘nudges’ alongside denser educational materials to cater to different stages of the user journey [20].

### Leveraging decentralization for cultural connection

The study validates the effectiveness of the decentralized communication model. State Government (Gujarat) materials scored significantly higher than Central Government (New Delhi) materials in cultural tailoring (Table 8) and linguistic clarity (Table 9). This confirms that state-level teams are adept at creating content that resonates with the local context, a core principle of Social Cognitive Theory [5]. The finding that vernacular (Gujarati) materials achieved the highest clarity scores supports the continued prioritization of regional languages for mass communication.

However, correlation analysis (Table 12) reveals a crucial nuance: cultural tailoring (*r* = 0.03) operates independently of scientific breadth. This implies that while state agencies excel at adapting the form of communication (language, visuals), this does not automatically translate to adapting the substance (scientific depth). True localization requires more than translation; it requires reframing scientific concepts into local cultural metaphors. A collaborative model where central agencies provide the rigorous scientific core and state bureaus adapt the delivery could combine technical accuracy with deep cultural resonance.

### Optimizing formats for the digital age

The comparative efficacy analysis (Fig. 2; Table 7) highlights the potential of dynamic media. Video materials performed strongly in both Global Clarity and the Action Index. Notably, the analysis of video duration (Table 3) found that short videos (< 1 min) were statistically as effective as longer formats in terms of clarity (*p* = 0.482) and actionability (*p* = 0.803). This finding supports a strategic expansion into ‘snackable’ content (e.g., YouTube Shorts, Instagram Reels) to capture attention in a crowded media landscape [21].

Despite this potential, the corpus remains 84.5% print-based (Table 1), with posters constituting the vast majority (68.0%) of print outputs (Table 2). While posters are excellent for passive visibility, the relatively low proportion of take-home materials like leaflets (8.5%) limits opportunities for detailed patient education. Recent experiences with COVID-19 communication in India demonstrated the high reach of short-form digital content [22]. Furthermore, digital platforms like WhatsApp have been shown to be critical vectors for health information dissemination in India, necessitating content designed for rapid sharing [23]. Gradually rebalancing the portfolio to include more digital-first assets could help the NTEP leverage these platforms to reach younger demographics.

### Target audience and educational depth

The segmentation analysis (Table 11) provides evidence of a stratified communication strategy. Materials designed for patients and caregivers exhibited significantly higher Total Behavioral Breadth compared to those for the general public (*p* = 0.041). This suggests the system correctly identifies that patients require greater educational depth (complex regimens, side effects) while the general public requires brevity. However, the lack of pre-testing evidence (Table 6) raises the question of whether this added depth is accessible or overwhelming. Without user validation-a mandatory step in the WHO COMBI framework [9] -there is a risk that ‘comprehensive’ materials may exceed the health literacy of the average patient.

### Strengths and limitations

This study’s primary strength is its comprehensive scope, integrating seven distinct behavioral frameworks to assess a large, multi-format corpus across state and central sources. The methodology moves beyond simple readability checks to evaluate the behavioral fidelity of the materials. However, the study is limited by its desk-based design; it assesses the content of the materials but not their impact on actual patient behavior or knowledge. Furthermore, this study evaluated *behavioral design fidelity* rather than *biomedical accuracy*; we did not assess the clinical validity of the medical advice provided. As the coding was performed by researchers, the results reflect expert evaluation which may differ from the lay audience’s perception. Future studies should incorporate qualitative audience reception analysis to address this potential evaluator bias. Additionally, the exclusion of offline-only materials (e.g., street plays) may limit the generalizability of findings to the full spectrum of IEC activities. Finally, the analysis of audio materials was constrained by a small sample size (*n* = 4), necessitating cautious interpretation of audio-specific findings.

### Practice and policy implications

To align TB communication with India’s ambitious elimination goals, the following evidence-based shifts are recommended for the State TB Cell and National policymakers:

1. Formalize Feedback Loops:

Implement a rapid ‘pre-test protocol’ for new creatives. Ensuring that a sample of the target audience reviews key messages before release will help validate clarity and cultural appropriateness.

2. Expand the Preventive Narrative:

Gradually increase the proportion of creatives focusing on TPT, Latent TB, and Vaccination to support the National Strategic Plan’s elimination goals.

3. Enhance Digital Engagement:

Allocate resources toward the production of high-quality, short-form (< 1 min) videos that are optimized for social media sharing, capitalizing on their proven clarity and actionability.

4. Adopt Enabling Messaging:

Enriched scripts that acknowledge patient challenges (e.g., side effects, costs) and offer practical solutions can improve self-efficacy and treatment adherence.

5. Collaborative Localization (Global Relevance):

Encourage a model where editable design files are shared with districts, allowing for deep localization of visuals and dialects while maintaining scientific accuracy. This ‘Core-Adaptation’ model is scalable to other federalized health systems in LMICs.

### Reproducibility and data transparency

The complete item-level dataset is publicly available in an open-access repository (Figshare), and the full codebook and reliability statistics are provided as Supplementary Files. This allows independent replication and reuse of the framework for comparative analyses of health communication quality.

## Conclusions

The TB communication landscape in Gujarat is characterized by high institutional authority, strong clarity, and effective cultural adaptation at the state level. However, the current strategy remains predominantly print-based, biomedical in focus, and directive in tone. To transition from TB control to elimination, future communication efforts must pivot toward enabling, empathetic messaging that addresses social barriers and leverages the power of short-form digital media. By institutionalizing pre-testing and expanding the narrative to include prevention and stigma reduction, the program can ensure that every message not only reaches the public but is designed with the behavioral fidelity to empower action.

## Supplementary Information

Supplementary Material 1.

Supplementary Material 2.

Supplementary Material 3.

Supplementary Material 4.

## Data Availability

The dataset generated and analysed during the current study is publicly available in the Figshare repository at: DOI: 10.6084/m9.figshare.31095055. The study codebook, reporting checklist, and inter-rater reliability outputs are provided as Supplementary Files with this manuscript. The materials analyzed are publicly available documents released by the Government of India and the Government of Gujarat for public health awareness.
