# Supplementary material for: Every message counts: scientific analysis of tuberculosis communication materials in Gujarat
Source: BMC Public Health. 2026 May 11;26:1955. doi: 10.1186/s12889-026-27648-y (PMC13295647; doi:10.1186/s12889-026-27648-y)
Supplement: Supplementary file 1 — Supplementary Material 1. [file 12889_2026_27648_MOESM1_ESM.docx]

**Table S2. Inter-Rater Reliability Audit (Summary of inter-rater reliability statistics for the subset of double-coded IEC materials (n=38).**

| **Measurement Domain** | **Statistical Metric** | **Value** | **Interpretation** |
| --- | --- | --- | --- |
| **Binary Checklist Items (Content)** |  |  |  |
| COM-B Model (6 items) | Cohen’s Kappa 𝛋 | 0.84 | Strong Agreement |
| Health Belief Model (6 items) | Cohen’s Kappa 𝛋 | 0.89 | Strong Agreement |
| Theory of Planned Behavior (3 items) | Cohen’s Kappa 𝛋 | 0.82 | Strong Agreement |
| Social Cognitive Theory (4 items) | Cohen’s Kappa 𝛋 | 0.86 | Strong Agreement |
| Transtheoretical Model (3 items) | Cohen’s Kappa 𝛋 | 0.91 | Almost Perfect |
| WHO COMBI & CDC Index (18 items) | Cohen’s Kappa 𝛋 | 0.94 | Almost Perfect |
| **Average (Total Behavioral Breadth)** | **Mean kappa 𝛋** | **0.88** | **Strong Agreement** |
|  |  |  |  |
| **Qualitative Ratings (Execution)** |  |  |  |
| Global Clarity Score (1–5 Scale) | ICC (2,1)* | 0.92 | Excellent Reliability |
| Global Cultural Adaptation Score (1–5 Scale) | ICC (2,1)* | 0.89 | Good to Excellent |

Note: ICC (2,1) = Two-way mixed effects, absolute agreement, single measures model. Interpretation benchmarks: 0.60–0.79 (Moderate/Strong), 0.80–0.90 (Strong), >0.90 (Almost Perfect). Discrepancies were resolved via consensus meeting.
