# Supplementary material for: Every message counts: scientific analysis of tuberculosis communication materials in Gujarat
Source: BMC Public Health. 2026 May 11;26:1955. doi: 10.1186/s12889-026-27648-y (PMC13295647; doi:10.1186/s12889-026-27648-y)
Supplement: Supplementary file 2 — Supplementary Material 2. [file 12889_2026_27648_MOESM2_ESM.docx]

# 40-Point Scientific Assessment Codebook & Scoring Guide

**Description of data:** This supplementary file details the comprehensive 40-item codebook used to evaluate the behavioral fidelity and scientific quality of TB IEC materials. It outlines the specific constructs derived from seven behavioral theories (COM-B, HBM, TPB, SCT, TTM, WHO COMBI, CDC Index), the criteria for binary scoring (Presence/Absence), and the rubrics for the supplementary 5-point Likert scales used to assess visual clarity and cultural adaptation.

## Part A: The 40-Point Behavioral Breadth Checklist

**Scoring Protocol:**

- **1 (Present):** The construct is explicitly mentioned, visually depicted, or audibly stated.
- **0 (Absent):** The construct is missing, vague, or requires significant inference.

| **Item #** | **Theoretical Framework** | **Domain / Construct** | **Specific Assessment Criteria (Evidence to look for)** |
| --- | --- | --- | --- |
| **1** | **COM-B** | Physical Capability | Does it demonstrate a skill? (e.g., showing how to wear a mask, how to dispose of sputum, how to cover a cough). Simple instruction without demo = 0. |
| **2** | **COM-B** | Psychological Capability | Does it provide factual knowledge? (e.g., causes of TB, specific symptoms, transmission routes). |
| **3** | **COM-B** | Physical Opportunity | Does it mention resources/access? (e.g., "Free testing," location of centers, "call 1800..."). |
| **4** | **COM-B** | Social Opportunity | Does it leverage social norms? (e.g., showing family support, community leaders, or depicting TB testing as a socially responsible act). |
| **5** | **COM-B** | Reflective Motivation | Does it appeal to intent/planning? (e.g., "Decide to get tested today," "Make a plan to visit the doctor"). |
| **6** | **COM-B** | Automatic Motivation | Does it trigger emotion? (e.g., Fear of lung damage, Hope of a cure, Pride in being healthy). |
| **7** | **HBM** | Perceived Susceptibility | Does it highlight personal risk? (e.g., "Anyone can get TB," "TB does not discriminate"). |
| **8** | **HBM** | Perceived Severity | Does it emphasize consequences? (e.g., "TB can be fatal," "It spreads to your family"). |
| **9** | **HBM** | Perceived Benefits | Does it highlight the positive outcome? (e.g., "Treatment cures you," "Get back to work," "Live a healthy life"). |
| **10** | **HBM** | Perceived Barriers | Does it address obstacles? (e.g., "Drugs are free" [cost barrier], "Side effects are manageable" [physical barrier]). |
| **11** | **HBM** | Self-Efficacy | Does it build confidence? (e.g., "You can defeat TB," testimonials from survivors saying "I did it"). |
| **12** | **HBM** | Cues to Action | Is there a specific trigger? (e.g., Helpline number, QR code, Website URL). |
| **13** | **TPB** | Attitude | Does it frame the behavior as positive/smart? (e.g., "Testing is a wise choice"). |
| **14** | **TPB** | Subjective Norms | Does it reference what others think? (e.g., "Your neighbors will respect you for being safe"). |
| **15** | **TPB** | Perceived Control | Does it emphasize individual agency? (e.g., "The power to end TB is in your hands"). |
| **16** | **SCT** | Observational Learning | Is there a role model performing the behavior? (e.g., Celebrity, Politician, or relatable peer character). |
| **17** | **SCT** | Reinforcement | Are there rewards mentioned? (e.g., Rs 500 DBT, Nutrition Baskets, improved health status). |
| **18** | **SCT** | Self-Efficacy (SCT) | *Scored same as HBM Q11. (Confidence in execution).* |
| **19** | **SCT** | Reciprocal Determinism | Does the environment support the person? (e.g., Workplace ventilation, cleaner air, supportive infrastructure). |
| **20** | **TTM** | Pre-Contemplation | Focus on awareness? (e.g., "Do you have a cough?" - targeted at those unaware of the problem). |
| **21** | **TTM** | Action | Focus on steps? (e.g., Dosage schedule, clinic timings - targeted at those ready to act). |
| **22** | **TTM** | Maintenance | Focus on adherence? (e.g., "Complete the course," "Don't stop medicine"). |
| **23** | **WHO COMBI** | Programme Objective | Is a specific goal stated? (e.g., "TB Mukt Bharat," "End TB by 2025"). |
| **24** | **WHO COMBI** | Behavioral Objective | Is the specific behavior clear? (e.g., "Get Tested" is clear; "Be Aware" is vague). |
| **25** | **WHO COMBI** | Audience Segmentation | Is it tailored for a specific group? (e.g., Pregnant women, Smokers, Children). Generic = 0. |
| **26** | **WHO COMBI** | Strategy Mix | Does it mention a campaign/strategy? (e.g., "Jan Andolan," "Ni-kshay Mitra"). |
| **27** | **WHO COMBI** | Channel Mix | Does it cross-reference other media? (e.g., A poster mentioning a radio show or app). |
| **28** | **WHO COMBI** | Pre-testing Evidence | Is there explicit evidence/text stating the material was field-tested? (e.g., "Tested with..."). |
| **29** | **WHO COMBI** | Implementation Plan | Are timelines/rollout details mentioned? (Rare in single posters). |
| **30** | **WHO COMBI** | Monitoring Indicators | Is there a way to track success? (e.g., "Give a missed call to register"). |
| **31** | **CDC Index** | Main Message Placement | Is the key message in the top 30% (Print) or first 5 seconds (Video)? |
| **32** | **CDC Index** | Call to Action | Does it use an active verb? (e.g., "Go," "Call," "Wear"). Passive voice ("TB is curable") = 0. |
| **33** | **CDC Index** | Language Simplicity | Is the language jargon-free? (e.g., Uses "Cough test" instead of "Sputum Microscopy"). |
| **34** | **CDC Index** | Information Chunking | Is text broken into bullets/sections with headers? (Walls of text = 0). |
| **35** | **CDC Index** | Behavioral Directions | Are there numbered steps? (1, 2, 3...). |
| **36** | **CDC Index** | Numbers Meaningful | Are statistics explained with context? (e.g., "1 in 4 people" instead of "25%"). |
| **37** | **CDC Index** | Visual Congruence | Do images match the text? (e.g., Text says "Fever," image shows thermometer). |
| **38** | **CDC Index** | Risk Explanation | Is the threat visually or textually clear? (e.g., Lungs depicted as damaged). |
| **39** | **CDC Index** | Next Steps | Are contact details (Address/Phone) clearly visible? |
| **40** | **CDC Index** | Evidence/Credibility | Are official logos (Ashok Stambh, NTEP, G20) present? |

## Part B: Qualitative Assessment Scales

These items were scored on a 5-point Likert scale to assess the *quality of execution*, independent of the binary content score.

### 1. Global Clarity Score

*Measures visual accessibility, typography, and ease of reading.*

- **1 (Very Poor):** Cluttered layout, "wall of text," font too small to read from distance, low contrast, audio inaudible.
- **2 (Poor):** Confusing layout, mixed fonts, key message buried.
- **3 (Average):** Standard government template. Readable but text-heavy or unengaging.
- **4 (Good):** Clear hierarchy (Headline > Body), good contrast, simple fonts, professional audio mix.
- **5 (Excellent):** "Picture Superiority" (Image tells the story without text), minimalist design, high-impact visuals, perfect production value.

### 2. Global Cultural Adaptation Score

*Measures contextual relevance to the Gujarat setting.*

- **1 (Generic/Foreign):** Western stock photos (white doctors/patients), English-only text, non-Indian settings.
- **2 (Generic Indian):** North Indian context (Hindi text only, Delhi-centric backgrounds), generic Indian vectors.
- **3 (Translation):** Gujarati text present, but reads like a direct translation from English/Hindi. Standard Indian photos.
- **4 (Adapted):** Gujarati text with some local idioms. Photos show Indian patients in generic settings.
- **5 (Highly Localized):** Native Gujarati (Kathiawadi/Surti dialects). Visuals feature local dress (Saree styles/Paaghadi), local architecture (Pol houses/Gamdu), and specific local cultural references (e.g., referencing local festivals).

## Part C: Sub-Index Calculation Guide

- **Total Behavioral Breadth:** Sum of items 1–40 (Range: 0–40).
- **HBM Index:** Sum of items 7–12 (Range: 0–6).
- **COM-B Index:** Sum of items 1–6 (Range: 0–6).
- **CDC Clarity Index:** Sum of items 31–40 (Range: 0–10).
- **Action Index:** Sum of items 12 (Cues) + 32 (Call to Action) + 39 (Next Steps). (Range: 0–3).
