# Supplementary material for: Every message counts: scientific analysis of tuberculosis communication materials in Gujarat
Source: BMC Public Health. 2026 May 11;26:1955. doi: 10.1186/s12889-026-27648-y (PMC13295647; doi:10.1186/s12889-026-27648-y)
Supplement: Supplementary file 3 — Supplementary Material 3. [file 12889_2026_27648_MOESM3_ESM.docx]

**Table S1. STROBE Statement-Checklist of items that should be included in reports of cross-sectional studies**

| **Item No** | **Recommendation** | **Reported on Page/Section** |
| --- | --- | --- |
| Title and Abstract |  |  |
| 1 | (a) Indicate the study’s design with a commonly used term in the title or the abstract | Title & Abstract |
|  | (b) Provide in the abstract an informative and balanced summary of what was done and what was found | Abstract |
| Introduction |  |  |
| 2 | Explain the scientific background and rationale for the investigation being reported | Introduction |
| 3 | State specific objectives, including any prespecified hypotheses | Introduction (Final Paragraph) |
| Methods |  |  |
| 4 | Present key elements of study design early in the paper | Methods > Study Design |
| 5 | Describe the setting, locations, and relevant dates, including periods of recruitment, exposure, follow-up, and data collection | Methods > Study Design |
| 6 | (a) Give the eligibility criteria, and the sources and methods of selection of participants | Methods > Materials |
| 7 | Clearly define all outcomes, exposures, predictors, potential confounders, and effect modifiers | Methods > Codebook Development |
| 8 | For each variable of interest, give sources of data and details of methods of assessment (measurement). Describe comparability of assessment methods if there is more than one group | Methods > Coding Process |
| 9 | Describe any efforts to address potential sources of bias | Methods > Reliability Assessment |
| 10 | Explain how the study size was arrived at | Methods > Sampling Strategy |
| 11 | Explain how quantitative variables were handled in the analyses | Methods > Data Analysis |
| 12 | (a) Describe all statistical methods, including those used to control for confounding | Methods > Data Analysis |
|  | (b) Describe any methods used to examine subgroups and interactions | Methods > Data Analysis |
|  | (c) Explain how missing data were addressed | Methods > Data Analysis |
| Results |  |  |
| 13 | (a) Report numbers of individuals at each stage of study (e.g., numbers potentially eligible, examined for eligibility, confirmed eligible, included in the study, completing follow-up, and analysed) | Results > Corpus Characteristics |
|  | (b) Give reasons for non-participation at each stage | Methods > Sampling Strategy |
| 14 | (a) Give characteristics of study participants (e.g., demographic, clinical, social) and information on exposures and potential confounders | Table 1 |
| 15 | Report outcome data in sufficient detail to allow quantitative analysis | Tables 4, 5, 6 |
| 16 | Report main results (e.g., unadjusted estimates and, if applicable, confounder-adjusted estimates and their precision). Report 95% Confidence Intervals. | Tables 7, 8, 9, 10 |
| 17 | Report other analyses done-e.g., analyses of subgroups and interactions, and sensitivity analyses | Results > Comparative Efficacy |
| Discussion |  |  |
| 18 | Summarize key results with reference to study objectives | Discussion > Paragraph 1 |
| 19 | Discuss limitations of the study, taking into account sources of potential bias or imprecision | Discussion > Strengths and Limitations |
| 20 | Give a cautious overall interpretation of results considering objectives, limitations, multiplicity of analyses, results from similar studies, and other relevant evidence | Discussion > Practice and Policy Implications |
| 21 | Discuss the generalisability (external validity) of the study results | Discussion > Strengths and Limitations |
| Other Information |  |  |
| 22 | Give the source of funding and the role of the funders for the present study and, if applicable, for the original study on which the present article is based | Declarations > Funding |
